# ‘Ingredients’ of a supportive web of caring relationships at the end of life: findings from a community research project in Austria

**DOI:** 10.1111/1467-9566.12738

**Published:** 2018-04-27

**Authors:** Klaus Wegleitner, Patrick Schuchter, Sonja Prieth

**Affiliations:** ^1^ Department for Palliative Care and Organisational Ethics Institute of Pastoral Theology University of Graz Graz Austria; ^2^ Institute of Palliative Care and Organisational Ethics University of Klagenfurt Vienna Austria; ^3^ Tyrolean Hospice Association Innsbruck Austria

**Keywords:** community care, end of life care, palliative care, public health, inequalities/social inequalities in health status, social capital

## Abstract

In accordance with the pluralisation of life plans in late modernity, the societal organisation of care at the end of life is diverse. Although the public discourse in western societies is dominated by questions about optimising specialised palliative care services, public health approaches, which take into account the social determinants and inequalities in end‐of‐life care, have gained in importance over the last decade. Conceptual aspects, dimensions of impact and benefit for the dying and their communities are well discussed in the public health end‐of‐life care research literature. Our research focuses on the preconditions of a supportive caring web in order to understand how communities can build on their social capital to deal with existential uncertainty. As part of a large‐scale community research project, we carried out focus groups and interviews with community members. Through dispositive analysis, we generated a set of care‐web ‘ingredients’, which constitute and foster a caring community. These ‘ingredients’ need to be cultivated through an ongoing process of co‐creation. This requires: (i) a focus on relationships and social systems; (ii) the creation of reflective spaces; and (iii) the strengthening of social capital, and d) the addressing of inequalities in care.

## Introduction

What does end‐of‐life care mean in late modern western societies? Generally speaking, this subject deals with the collective coping process with the universal and existential uncertainty of dying in – in principle – precarious and fragile liquid modernity (Bauman 2013). Plural life plans in secular societies imply plural and diverse understandings of ‘good’ dying and also of ‘appropriate’ approaches, practices and interventions in end‐of‐life care (Clark *et al*. 2017, Zaman *et al*. 2017). Therefore, the public discourse about the ethics of dying and end‐of‐life care is increasing continuously in western societies.

In the second half of the 20th century, research and reflections about death and dying in care institutions (Elias 1982, Feifel 1959, Foucault 1973, Glaser and Strauss 1966, Kübler‐Ross *et al*. 1972, Saunders 1960, Sudnow 1967) led to a critical analysis of medicalised, socially hidden and tabooed dying in hospitals and in society in general.

In conjunction with this and partly independent from it, the process of institutionalisation and medicalisation of dying has evoked counter movements, such as the hospice movement and palliative care (Saunders 2001, WHO 2002). In many regions, this marked the beginning of a new era of specialised end‐of‐life care (Centeno *et al*. 2016). The international palliative care development is a success story. In many countries, it raised professionalised care for the dying and their families to a new level of humanity. However, it also contributed to the reproduction and continuation of the institutionalisation of end‐of‐life care (Randall and Downie 2006), while it has not yet overcome the global inequity of access to palliative care (Knaul *et al*. 2015).

From the late 1990s onwards, the counter movements that followed in end‐of‐life care were directed against the professionalisation and expertocratisation of dying. We would – ideal‐typically – interpret this as trends which aspire to: (i) the individualisation of end‐of‐life care, in terms of autonomously planning one′s dying – maybe as last ‘project’ in the course of life (through patient decree, advance care planning, physician‐assisted suicide, euthanasia, etc.); and (ii) the democratisation (and social embeddedness of end‐of‐life care (through strengthening one′s ability to handle and cope with care situations, promoting death literacy, social integration of dying and death, etc.).

In that respect, the new public health approach has gained in importance in end‐of‐life care over the last decade. A wide range of practices and international efforts are now incorporating principles of health promotion into end‐of‐life care (Kellehear 1999, 2005, Sallnow *et al*. 2012), according to the Ottawa Charter (WHO 1986). These initiatives and projects are often described using the phrase ‘compassionate communities’ (Wegleitner *et al*. 2015aa). More generally speaking, the new public health approach in end‐of‐life care ‘can be understood as a series of social efforts by communities, governments, state institutions and social or medical care organisations that aim to improve health and wellbeing in the face of life‐limiting illness’ (Sallnow *et al*. 2016: 201). Although it remains a marginal discourse in the field of palliative care as a whole (Zaman *et al*. 2017), the public health approach represents a paradigmatic shift in palliative care policy and practice; a departure from the models of delivering services of care towards a community and civic society‐based approach in end‐of‐life care.

This public health perspective on end‐of‐life care takes into account the meaning of social determinants and social‐structural environment in improving health and wellbeing, as well as in (re)producing inequalities in health care in all phases of life. In doing so, these initiatives have sparked an awareness about an,ecological care holism’ (Kellehear 2005: 49f) in the – so far – predominantly individual, face to face, care‐driven practice and theory of palliative care. Reflecting this public health end‐of‐life care progresses from a more sociological perspective shows promising interconnections with research on the impacts of social relationships on health (House *et al*. 1988, Umberson and Karas Montez 2010) and mortality (Holt‐Lunstad *et al*. 2010) as well as of social networks on health (Reeves *et al*. 2014) and mortality; particularly in low‐income countries (Santini *et al*. 2015). The relation of social capital and social cohesion with health and mortality in the family (Álvarez *et al*. 2017), in the neighbourhood (Lochner *et al*. 2003; Ziersch *et al*. 2005) and in the community (Kawachi *et al*. 1997, Kawachi 1999, Álvarez and Romani 2017) represents a key issue therein. In addition, dimensions and factors of social justice (Ruger 2004) and health inequalities (Bernard *et al*. 2007, Dahl and Malmberg‐Heimonen 2010) are of particular importance in end‐of‐life care and should therefore be addressed more explicitly.

So far, the conceptual framework of public health end‐of‐life care has been widely discussed (Abel *et al*. 2013, Abel and Kellehear 2016, Kellehear 1999, Rosenberg and Yates 2010, Sallnow and Paul 2015). First attempts to determine the impact of public health end‐of‐life care approaches suggest that engaging communities in health matters leads to improvements in health and wellbeing. It also promotes the development of community capital (Sallnow *et al*. 2016). Moreover, some studies show that these interventions have multiple beneficial effects on the quality of end‐of‐life care, on improving social capital and on one′s ‘death literacy’ (Horsfall *et al*. 2012, McLoughlin *et al*. 2015, Noonan *et al*. 2016, Rosenberg *et al*. 2015).

Debbie Horsfall and her colleagues (2015) have addressed the question of ‘good’ end‐of‐life care based on an ecological view of caring with regard to care ethics. Central to this is ‘that the dying person and their caring networks exist within a complex web of social‐environmental relationships where the knowledge, skills, values, attitudes and beliefs of network members are influenced’ by various factors (Horsfall *et al*. 2015: 7). Existing relationships, local informal and formal care resources, and a communal living culture that includes social norms and expectations as well as shared knowledge and experiences of care and ecological conditions all affect local end‐of‐life care practice and culture at an individual, collective and communal level.

Therefore, complementary to conceptual framing and aspects of impact and benefit, we associate our research with this understanding of an ecology of care and direct our attention particularly towards the qualitative characteristics of a supportive caring web in the community at the end of life.

As part of a more comprehensive project in Austria, we investigated the following questions through participatory research: What forms of relationships and practices do people concerned experience as ‘caring’? Building on this, what constitutes a network or ‘web’ of caring relationships on a community level? Which general conclusions can be drawn from our research to understand the key elements that encourage supportive care webs at the end of life?

## Researching local care needs and practices: a multi‐perspective and circular process

The study framework has been a large‐scale community‐based participatory research (Minkler and Wallerstein 2003; Hockley *et al*. 2013, Maiter *et al*. 2008) project called ‘caring community in living and dying’. Undertaken in Tyrolean community of Landeck, it aimed to strengthen networks and solidarity in the community, in order to support older and vulnerable people as well as family caregivers. The awareness about existential questions concerning vulnerability, dying, death and loss and about the distribution of care work (Tronto 2013) should be increased. Given the fact that the project process reached only a part of the citizens and the municipalities’ population is diverse, and therefore cannot be understood as a homogenous community, the project intervention merely opened up potentials of change and established traces for how to improve the local care web. The two‐year duration of the project was divided roughly into three phases.

In this article, we focus on findings from the first phase, the objective of which was to understand the local care practice and culture with its resources, deficits, and peculiarities from a variety of different perspectives. Because of local needs and actual questions raised in the region, the project and consequently the survey itself concentrated on the situation of family carers.

### Sample

The following focus groups and individual interviews were undertaken:

- Three focus groups with family carers in different stages of their caring process: home care situation in the past (n = 4), current care situation at home (n = 3), and home care situation in the past and current care by a nursing home (n = 4). All participants were women.
- One focus group (n = 6, all women) comprising hospice volunteers in the region.
- One focus group (n = 3, all women) comprising coordinators of self‐help groups and voluntary work for family carers.
- Individual interview with the local undertaker (a woman).
- Individual interview with the local (Catholic) priest.
- One focus group with community general practitioners (n = 4, one woman).
- One focus group with members of the ambulatory nursing care team (n = 4, all women).

This sampling depended on our ‘door‐openers’ from local hospice work. This entailed: (i) a focus on the situation of caring relatives; and (ii) only reaching those who had been in contact with informal (volunteers, self‐help groups) or formal caregivers.

### Interview guidelines and data analysis

The methodological approach for our qualitative survey was broadly based on ‘dispositive research’ as developed by Bührmann and Schneider (2015). The concept of a ‘dispositive’, as introduced by Foucault and operationalised for empirical research by Bührmann and Schneider, refers to a ‘heterogeneous ensemble consisting of discourses, institutions, architectural forms, regulatory decisions, laws, administrative measures, scientific statements, philosophical, moral and philanthropic propositions’ that make up a system of relations (Foucault 1980: 194). This theoretical construct allows us to consider and analyze the complex and dynamic system of elements affecting a social issue. Heuristically we talked of the ‘order of care’. This dispositive research framework served as a basis for developing interview guidelines as well as a tool and a general heuristic framework for data analysis.

The validity of data analysis has been assured using different methodological measures. A general structured reading and analysis of the transcription roughly adhered to the approach for researching lived experience as developed by Lindseth and Norberg (2004).

An initial open reading provided a ‘naïve’ understanding in the first step. In the second reading – structural analysis – we tried to combine the open approach for capturing the meaning of lived experience as proposed by Lindseth and Norberg with a more detailed line of questioning derived from dispositive analysis.

In this step, the texts were read by three researchers, each of them also focusing more closely on one aspect of the dispositive framework content (forms of care knowledge, forms of care practices, and care objects). This facilitated a form of investigator triangulation (Archibald 2016, Denzin 1978). In two data analysis workshops, perspectives were exchanged, emerging key themes were retained, meaning units and quotations were identified and chosen for feedback and ongoing work continued with people in the community. In addition to this, a reflective workshop with six other researchers broadened the analytical and interpretative potential.

The data collected were prepared for feedback to the community and for further work in the project, where insights, key issues and quotations served as stimulus for e.g. in‐depth discussions, future workshops, and a public forum in the town hall.

## The web of caring relationships: qualities and attributes as ‘ingredients’

Based on the dispositive approach, our analysis aimed to reflect the local care practice from different perspectives and to include individual, organisational, network‐oriented and communal (political) dimensions relating: (i) to forms of care‐knowledge and discourses (with its power relations); (ii) to care‐acting (e.g. care roles, values, competencies); and (iii) to ‘care‐artefacts’ (material environment, meaningful objects). As a part of this dispositive we could identify the main qualities and attributes of a supportive local care practice. During the course of the analysis, it became clear that these qualities could neither be selectively assigned to individuals nor to organisations or to specific social roles. They are neither attributes of specific care networks nor of specific social relationships. Therefore, we decided to speak of a ‘supportive care culture’ or of a ‘supportive web of caring relationships’. In this sense, a ‘web’ represents a hybrid formation that integrates individual and collective, as well as cultural (in the sense of shared values, understandings and practices) and structural dimensions of care.

Against this background we interpreted the key qualities and attributes of a ‘supportive care web’ – as broadly as possible – as ‘ingredients’. The term ‘ingredients’ should not evoke the idea of a recipe or a checklist. It refers – metaphorically speaking – to the original idea of good cuisine and home cooking, where the available raw products need to be creatively combined and adjusted to the specific table fellowship. Sometimes, not everything is available and dosages may vary. Most important: high‐quality, organic raw products have to be carefully cultivated. Right intuition and feelings about the collective and individual tastes are necessary and the dish is experienced as a whole. Similarly, care is experienced as a whole, as a synergy, as a complex interplay of many ingredients.

Accordingly, in the case of a ‘care web’: (i) it is not possible to determine from the outset who contributes a certain ‘ingredient’ to the care web and in which specific social role or structure, as it is a social process of co‐creation; and (ii) the required ‘dosage’ may differ from case to case. For this reason, we can understand the ‘ingredients’ as abstract entities, which come to life through individuals and organisations in their different roles, networks and as a shared quality of the community, which strengthens social capital in care situations.

In the following, we describe the ‘ingredients’ by presenting relevant extracts from our focus group and interview data. In the subsequent discussion, we will set out to connect the insights and questions that emerged to theory building in relation to social capital, care ethics and care politics.

### Contributing specific competencies

Of course, all our participants identified members of professional care services as important partners in care, due to their skills and competencies. There is certainly a need for specialist knowledge, in the sense of professional medical, nursing and social work or for example the expertise and work of the notary (on inheritance matters) or the undertaker. This expert knowledge is obviously what distinguishes healthcare professionals from other people involved. However, specialist knowledge is not only the property of specialists themselves. To a certain, even if usually very limited, extent, specialist discourse or aspects of it, are appropriated by others. Family carers, for example, have often learned how to deal with catheters, suction systems or the side effects of drugs. In some cases, family carers have attended training courses in nursing techniques.

The major part of ‘traditional’ palliative care has focused on developing specialist competencies and institutions. The subsequent ‘ingredients’ demonstrate the relativity of this profession‐centred end‐of‐life care approach and the necessity of developing informal resources, as well as the interplay between professional and lay dimensions of care at the end of life (cf. also Horsfall *et al*. 2013).

### Sharing wisdom of life

Specialist knowledge and professional practice by themselves are not enough. Life experience, of oneself or others, is another significant knowledge in terms of end‐of‐life care.

Some community members mentioned that people, who have endured a period of illness or who require care themselves, seem to be particularly valuable to others. This is not because they can immediately give practical advice, but because they are considered as conversation partners, who can understand a suffering person in a deep and non‐superficial manner and, thus, are able to facilitate a new ‘understanding of life’ (Rehnsfeldt and Eriksson 2004) in an existential crisis. In the words of a woman, who cared for her mother:

One really doesn't talk about the dark times, for example, when one's nerves are on edge and you become anxious. A lot of people don't want to hear this, or are not able to understand it. But I do also have a friend, who looked after her sister for years, and she understands. I think that I can tell things to a person who themselves has experience – it is very different, they react completely differently. Others just don't want to know, or they just don't listen to you. That makes me feel as though I am burdening them. (FG, family carers, 1795 et seq.)

Our findings relating to the issue of shared wisdom and life experience confirm that caring relationships can be ‘existentially’ rewarding for the caregiver in the sense of personal growth, connectedness and getting in touch with the mysteries of life and death: ‘I had a further five days of being alone with him. It was a precious time, more intense than anything else in thirty years of marriage. I would not want to have missed this’. (FG, volunteers, 1428–30)

### Keeping an eye on each other

This issue emerged when considering the role of neighbours in care situations and revealed an ambiguity that is difficult to balance. On one hand, a caring culture implies the presence of a neighbourhood, in which people keep an eye on each other and know about one another. A ‘good’ neighbour knows when somebody is not doing well and assists with small gestures of help. On the other hand, knowing about one another might quickly entail some form of social control and gossip. Thus, the great art of caring among neighbours involves people knowing about each other but not placing constraints on each other's freedom and intimacy, and without generating false beliefs or confusing feelings of ‘guilt’ and ‘shame’ (‘What would our neighbours say?’).

It is the small everyday details of neighbourliness that are so important. For example, if I know that the lady living next door to me has health problems, I can say that ‘I'll shop for you if you write down what you need and I will bring it to you’. So it's the really little things that absolutely anyone can do that need supporting. Not as a matter of obligation, of course, but just simply thinking about one another, keeping eyes and ears open. I even cook soup for my grumpy neighbour when she is ill. I think to myself, ‘She is very old after all. What can one do?’’ (FG, coordinators, 1444 et seq.)

An expression, which gained a degree of popularity over the duration of the project, and which often served both as a starting point for reflection and as an illustration of wise and neighbourly social attentiveness, was coined by a building's caretaker: ‘When the flowers decorating the balcony are missing, it is a first sign of withdrawal by an elderly person from social life’ (quote from a project event for ‘neighbours and people ‘who keep an eye’ on the community’). This observation leads to an interesting question for further research: which signs – comparable to the missing flowers on the balcony – could prompt community members to find the courage to look after somebody?

### Sensitively gaining access to house and soul

Being dependent on others and having to accept somebody else's help (see also Harrop *et al*. 2014) can be experienced as humiliating or shameful. It is difficult to ‘let others in’ – both into one's ‘soul’ and life, as well as into one's home.

In this context, the role of professions, which are not health care‐related but have a caring function in the community was considered in one workshop and various discussions, interviews and other project events. This perspective reflects the philosophy of a caring community particularly clearly by focusing on care‐attentiveness not originating from the health care system, volunteers, neighbours or members of the family. One example of such a caring role involves hairdressers, whose core competency and practice is obviously to groom and cut hair. However, they also provide family carers with an opportunity to find some rest and relaxation. It is sometimes easier to give voice to what weighs a person down in situations like this, than within the (at least potentially) conflictual context of the family. A very pertinent example was reported to us by the above‐cited caretaker, who is responsible for many of the local residential buildings. An elderly woman living alone called him repeatedly to see about an allegedly defective heating system. Of course, this served as a pretext to interrupt the many hours of solitude and isolation and enjoy the presence of a friendly young man, living an active life. We can imagine that the barriers both in terms of effort and shame to calling directly for more formally organised help are high. Thanks to his caring wisdom and sensitivity in not limiting this contact to his core business, the caretaker has contributed to keeping open the doors to this elderly lady's house and – metaphorically speaking – to her soul. By doing so, he served as a bridge for community life. (cf. Walter 2017).

### Vicariously organising care

The only occasion, when family carers reported to us in *tears* and accompanied by outbursts of anger, was when they described their experiences with authorities and official bodies during the organisation of care.

‘And wherever you go, you have to fill out forms if you need something, pages of them. You have to give all your details. I think that is crazy. Why does everyone need to know? And then I put my signature in the wrong place because I am in a hurry, and have to start again from the beginning. You have the feeling they take you for a fool, or a first grader. Or you are told that you will get 150 euro support for a shower in the bedroom, but before you can get the support, you have to go there ten times for appointments and then they tell you ‘oops, you have almost missed the deadline!’ (FG, family carers, 580 et seq.)

This account by a family carer points out that dealing with bureaucracy is not only strenuous and complicated but also frequently humiliating. The process of organising and coordinating care is more than mere ‘case and care management’, and more than avoiding interface problems for providers of care services. There is a lot more at stake than just the management of complexity and access to state subsidies for care support.

For reasons like this, organising care and dealing with bureaucracy is a form of advocacy that guarantees one remains a full citizen and member of the community.

### Moderating care arrangements

All too often, the situation is familiar: One single person – usually female – bears the main burden of everyday caring obligations:

My mother has so many children … and now there is only one. (FG, family carers, 651 et seq.)

The social care role is normally ascribed and assumed without explicit awareness. It is taken for granted. Issues of a just distribution of care work, as they emerged in our findings, seem to be rarely discussed within families (and beyond).

We identified three conditions, which contribute to a just and sustainable care web: awareness (and anticipation) of social roles, neutral facilitation of family discussions, division of everyday care work between two or more family carers or, at least, regular relief and support for the main family carer. We subsume these conditions under the term ‘moderation’, since moderation refers to the facilitation of discussion, which implies knowing and guiding different emerging roles in social systems, as well as to the social virtue of moderation as defined by Montesquieu. The ‘inventor’ of the separation of powers described moderation as a virtue of a society that can avoid extreme imbalances by dividing power and distributing tasks fairly (cf. Derathé 1952).

### Enabling freedom from care (Sorglosigkeit)

The German term *Sorglosigkeit* – literally ‘carefreeness’ – conveys the positive message of being able to leave one's worries behind for a while and not having to care for or be concerned about anything for some time. A wise and just, supportive and comprehensive web of caring relationships does, in a certain sense, also organise its own opposite: interruption of care, distraction and pleasure. Nevertheless, family carers tend to feel guilty (sometimes caused by the expectations of others) at taking time for themselves or use their spare leisure time for errands or other duties:

She really needed an immense amount of support afterwards, because for at least two years she repeatedly said: ‘I didn't do enough, I didn't do enough, I didn't do enough, I should have done much, much more’. And one had to keep picking her up again. She didn't grieve so much because the sick person she cared for was really awful to her, but she still had feelings of guilt. (FG, coordinators, 133 et seq.)

The importance of others, who occasionally help carers take a break from caring and who liberate carers from unnecessary feelings of guilt, was highlighted in many of our interviews.

Just for once, not listening, not seeing, not thinking! (FG, family carers, 428 et seq.)

In our interpretation of these descriptions, we prefer the more radical term and idea of ‘freedom from care’ over the common topos of ‘self‐care’, which, at least potentially, leaves open the possibility that self‐care might serve as a means of caring for others (‘recharging batteries’). However, the positive side of freedom from care is untroubled enjoyment of e.g. going skiing, spending time with friends, going for long walks with the dog, going to a concert or making a commitment to creative activities like painting or pottery.

## Discussion

Findings about the ‘ingredients’ of a care web and a fundamental understanding of how these characteristics could be put in place contribute to: (i) public health end‐of‐life care discourse and practice; as well as to (ii) reflecting community care with regard to strengthening social capital and the role of care organisations, the political framework and the civil society.

We understand care as a societal practice with shared responsibility, embedded in a fluid, situational web of care relationships with specific ‘ingredients’, which needs to be cultivated through an ongoing process of co‐creation. This ecology of care requires: (i) a focus on relationships and social systems in care practices; (ii) the creation of reflective spaces; (iii) the strengthening of social capital; and (iv) the addressing of inequalities and power relations in care.

### Focus on (webs of) relationships, not on individuals

First of all, we should ask the following question: Even if we have a web of caring relationships with all the ‘ingredients’ mentioned here, who exactly are the persons cared for? In other words: who is in the ‘centre’ of the care web? A natural and general answer would place the suffering, dying or ill person, the person cared for in the proper sense, at the centre of the care web. It is, of course, quite reasonable to do so. Nevertheless, we would like to argue that our representations of a care web should not centre upon a single person, whether the ill or dying person or the family carer, but rather upon the relationship of them both (or three, or more). Despite many focus group participants being family carers, none of them described solely their personal situation but rather the difficult arrangement of home care in the relational interplay between themselves and their mother or father or other persons being cared for. The same was true for the other community members. This point of view is consistent with the ‘relational ontology’ of care, stressed by care ethicists (Conradi and Vosman 2016, Sander‐Staudt 2006). A relational ‘care epistemology’ represents an extremely important alternative draft to traditional care services (and their financing), which focus on individuals. Care ethics urge us to take into account the support of social systems and relationships. Our findings do not describe dimensions of direct care (assistance in activities of daily living, such as dressing, eating, washing, etc.) but the web of relationships that support the direct care relationship (see also Leonard *et al*. 2013). This demands the transformation of individual focused care services towards the organisation of care with a view to strengthen the ‘whole’ social capital of the community by enabling the situational co‐creation of care.

### Creating reflective spaces for developing collective care wisdom and creating a shared care culture

Such a ‘reorientation of services’ (WHO 1986) requires an appropriate ‘organisational culture’ of care. Organisational culture can only be developed, if opportunities for reflection and collective learning are provided, that help to understand and challenge its implicit and unconscious *basic* underlying assumptions that give the organisation its unique characteristic (Heimerl and Wegleitner 2013, Luhmann 2006, Schein 1987), such as reflecting on ‘relational ontology’ of care versus focus on individuals, professional care versus more hidden care practices of community members, professional versus lay knowledge and so on.

In addition to this rationale for creating reflective spaces, our findings show how people concerned (people cared for and their carers) contribute to the community in a deep sense of ‘sharing life experience and wisdom’: care relationships have been described as a mutual ethical learning process (Schuchter and Heller 2015) or as the power to bear witness to goodness in suffering (Arman 2007), to create a new understanding of life. Something is missing in a caring web or in a process of caring, if this dimension of caring wisdom fails to be cultivated and the creating of meaning in caring processes (Rehnsfeldt and Eriksson 2004) fails to be perceived.

More generally speaking, in order to create supportive webs of caring relationships, it is necessary to rediscover ‘lay knowledge’ (Popay *et al*. 1998) in many senses beyond the dominance of expert knowledge. Expressed in the words of dispositive analysts Bührmann and Schneider (2015), this implies enabling and organising inter‐discourses, which mediate in different ways between different experiences, expectations, and therefore life‐worlds and value orientations as well as between expert and knowledge and competencies of lay people.

In sum, these perspectives correspond with Joan Tronto's (2013) suggestion that a ‘caring society’ requires settings, where people can learn from and about the lives of others. To achieve this, organisations and communities need to provide opportunities for people to share existential experiences, reflect their practical care insights and the underlying assumptions of their shared care practice, and consider what is important to them, especially during critical phases of their lives.

### Strengthening social capital

Reflecting the relation between ‘care culture’ and the qualities of a social fabric, our perspective very much corresponds with the discourse of social capital (Kawachi 1999). Hence, a supportive ‘culture of care web’ needs the integration of Bourdieu's interpretation of social capital (embedment of individuals in social networks and their relationships among each other), Coleman's approach (which focuses on socio‐structural environment) and Putnam's understanding, who adds important qualitative dimensions, like sense of belonging, community collaborations, civic participation, trust and reciprocity (Álvarez and Romani 2017). Strengthening the communities’ social capital also means to promote a ‘robust’ civil society and vice versa (Rosa *et al*. 2010).

In this respect, our findings, *inter alia*, emphasise the importance of the neighbourhood in building social capital, including the somehow unexpected care‐roles of hairdressers, taxi drivers and others, who sensitively gain access to house and soul. According to a remarkable argument of Dörner (2012), the proper sphere, in which to learn empathy, attentiveness and care, might be seen in the neighbourhood (less so within the spheres of family or friends), since neighbours are to some extent unfamiliar. Consequently, the circle of caring has to be extended – but at the same time close enough to be able to care. Neighbours – in this sense – are not engaged in everyday care and are not deeply involved in intimate care relationships and as dialogue partners for the exchange of life wisdom. Still, they can offer small gestures of help that sometimes make a great difference, not least on a symbolical level with regard to solidarity in everyday social life. In the case of deaths occurring in the neighbourhood, Walter (1999: 123) outlined the potential that death provides a basis for relationships and community. By sharing existential and universal experiences like death, dying and loss, neighbouring networks strengthen their social cohesion and generate a sense of community.

Fostering sustainable webs of relationships and cultivating the ‘ingredients’ in end‐of‐life care requires the development of health promoting environments on various levels of society. Hence, a major effort is needed at the level of social policy to promote the underlying conditions, which facilitate social space and community‐oriented care practice. These ‘new forms’ of collective dealing with vulnerability, dying and loss must operate in an intersectional and integrated way, and provide people with an opportunity to participate in co‐creating care webs and benefit from it.

### Addressing inequalities and power relations in care

In that respect, care ethics enrich this perspective of co‐creating care in in a fluid, situational web of care relationships with specific ‘ingredients’ in highlighting the importance of reciprocal care relationships and a just distribution of care work. In doing so, the question of how societies organise care becomes part of a broader socio‐political framework and is closely linked to the question of a ‘good life’ – until the end – in general.

Our research has been confronted with the central questions of care ethics: care work is still women's work and, as such, is unequally distributed along gender lines (cf. Tronto 2013). While – in typical cases – the daughter‐in‐law does the everyday care work, others use their ‘passes out’ (Tronto 2013) of caring responsibilities based on questionable reasons such as: gender (‘It is not a man's task …’), workload (‘The children work and do not have the time …’), residence (‘The sister lives too far away …’), charity (‘He finances the care, so …’), inability to care (‘I cannot manage assisting my mother with intimate hygiene …’). Of course, these and other justifications could be sound reasons not to engage in everyday care and perhaps instead to provide support from a distance or to generally withdraw from care. The problem is, however, that in reality these issues of a just distribution of care work, as they emerged in our findings, seem to be rarely discussed within families and beyond. Many main family carers feel a regular relief from caring obligations to serve justice.

Another social and political core issue associated with power relations and serious asymmetries concerns dealing with bureaucracy – a situation, in which people in an existential crisis are confronted with, at worst, standardised, cold and unresponsive procedures.

Becoming carers for others pitches people into a precarious situation. The existential situation of family carers has been described in our data in various expressions as the ‘collapse of a world’ (Wegleitner *et al*. 2015ab). It often involves a profound disruption of one's familiar experience of everyday life and its routines, one's familiar self‐understanding and life orientation. One's capacity to solve problems becomes dramatically reduced. If behaviours and conditions complicate the organisation of care, they also spell humiliation, the inner structure of which has been famously described by Avishai Margalit. He describes a ‘decent society as a society that does not humiliate. More precisely, it is a society whose institutions do not humiliate people who depend on them’. (Margalit 1997: 147) Humiliating involves any sort of behaviour or condition that (symbolically) constitutes a sound reason for a person to consider one's self‐respect injured through the removal of a person's control over her/his life (or important elements of it) and thus their exclusion from the ‘Family of Man’ (Margalit 1997). Consequently, our research confirms the necessity of ethical and political considerations on a ‘caring bureaucracy’ (Bourgault 2017).

## Limitations

An important limitation of our study concerns the socio‐demographic characteristics of our participants. Sampling was not based on categories like gender, age, care situation, place of care, etc. but – following a participatory action research approach – depended on our ‘door‐openers’ from local hospice work. Thus, determining the general applicability of our findings remains subject to further research and discourse.
